# Keeping Things Positive: Affect as a Mediator between Physical Activity and Psychological Functioning

**DOI:** 10.3390/ejihpe13110171

**Published:** 2023-11-02

**Authors:** Aliakbar Foroughi, Nils T. Henschel, Hassan Shahi, Scott S. Hall, Lawrence S. Meyers, Kheirollah Sadeghi, Aliakbar Parvizifard, Klaus Boehnke, Serge Brand

**Affiliations:** 1 Department of Clinical Psychology, School of Medicine, Kermanshah University of Medical Sciences (KUMS), Kermanshah 6714415333, Iran; foroughi_2002@yahoo.com (A.F.); hassanshahi84@gmail.com (H.S.); khirollahsadeghi@yahoo.com (K.S.); aliakbarparvizifar@gmail.com (A.P.); 2 Social Development and Health Promotion Research Center, Health Institute, Kermanshah University of Medical Sciences (KUMS), Kermanshah 6714415333, Iran; 3 Bremen International Graduate School of Social Sciences, Constructor University, 28759 Bremen, Germany; nhenschel@constructor.university (N.T.H.); kboehnke@constructor.university (K.B.); 4 Department of Psychology, Faculty of Social Sciences, Razi University, Kermanshah 94Q4+6G3, Iran; 5 Department of Early Childhood, Youth, and Family Studies, Ball State University, Muncie, IN 47306, USA; sshall@bsu.edu; 6 Psychology Department, College of Social Sciences & Interdisciplinary Studies, California State University, Sacramento, CA 95819, USA; larrym@csus.edu; 7 Center for Affective, Sleep and Stress Disorders, Psychiatric Clinics of the University of Basel, 4002 Basel, Switzerland; 8 Division of Sport Science and Psychosocial Health, Department of Sport, Exercise and Health, Faculty of Medicine, University of Basel, 4002 Basel, Switzerland; 9 Substance Abuse Prevention Research Center, Kermanshah University of Medical Sciences (KUMS), Kermanshah 6714415333, Iran; 10 Sleep Disorders Research Center, Kermanshah University of Medical Sciences (KUMS), Kermanshah 6714415333, Iran; 11 School of Medicine, Tehran University of Medical Sciences (TUMS), Tehran 14166-34793, Iran; 12 Center for Disaster Psychiatry and Disaster Psychology, Psychiatric Clinics of the University of Basel, 4002 Basel, Switzerland

**Keywords:** physical activity, positive and negative affect, psychological well-being, psychological dysfunctioning

## Abstract

Higher physical activity is generally associated with more favorable psychological functioning. However, the role of positive and negative affect in such associations is unclear. Accordingly, this cross-sectional study explored whether affect mediated the relationship of physical activity with psychological well-being (PWB) and psychological dysfunctioning (PD). Young Iranian adults (N = 200) completed self-rating questionnaires covering physical activity, positive and negative affect, and proxies of PWB and PD. Regression analyses indicated that higher physical activity levels and higher positive and lower negative affect predicted proxies of PWB. The same (albeit in the opposite direction) applied to proxies of PD. Structural equation modeling indicated that positive and negative affect mediated the relationship between physical activity and PWB/PD. Accordingly, change in affect might be an important mechanism behind the association of physical activity and PWB/PD. Future research should further explore this across target populations and cultural contexts. Longitudinal and/or experimental studies are needed to disentangle causality.

## 1. Introduction

Numerous empirical studies have investigated the relationships between physical activity (PA) and psychological functioning [1,2,3,4,5,6,7,8]. However, the mechanism behind their connection remains unclear. As our review of literature demonstrates, positive and negative affect could mediate associations between PA and psychological functioning. It appears, however, that research on the interplay among affect, regular PA, and trait-like psychological functioning are scarce, especially with sensitivity to a broader cultural context. The current study aimed to address this research gap. Results can be of practical importance when promoting positive psychological outcomes with the use of PA.

### 1.1. Physical Activity and Psychological Functioning

In the present context, psychological functioning embraces the broad range of emotions and cognitions associated with executive functions for physical activity performance. Accordingly, psychological functioning, in a broad sense, can be categorized into two major domains: (1) positive/favorable/adaptive psychological functioning, referred to as psychological well-being (PWB) [9], and (2) negative/unfavorable/maladaptive psychological functioning, referred to as psychological disorders or psychological dysfunctioning (PD) [10]. Various cross-sectional, longitudinal, and intervention studies [2,11,12,13] provide support for a connection between regular PA and PWB for older adults [4], children [2], adults in general [1], adolescents [12,14], and employees in the workplace [15]. Higher PA levels also improved sleep quality and promoted PWB [6,16].

Cross-sectional and intervention research [17,18,19,20,21] has indicated that regular physical activity decreased depressive symptoms and anxiety and improved emotional regulation. PA also favorably influenced cognitive emotional processes in individuals with schizophrenia spectrum disorders [3] and children with autism spectrum disorder [5]. PA appeared to protect against stress-induced health problems [11] and symptoms of dementia [22].

Scholars provided several possible explanations as to why regular PA might have such favorable impact. First, it may increase self-esteem [23], promote feelings of mastery [24,25] and satisfaction with life [26,27], and may strengthen feelings of belonging and interpersonal relationships [28]. Regular PA also causes the development and release of new neural pathways in the brain [29,30]. Each of these outcomes arguably contribute to overall psychological health.

Interestingly, another line of research [31,32,33] indicated that PA consistently has a stronger association with PWB during leisure time compared to during work, travel, housework, and physical education (PE). White et al. [29] suggested that motivation plays a mediating role between PA and final personal outcomes. When people perform PA during leisure activities, they experience *positive affect* and perform more PA because of intrinsic motivation. On the other hand, PA tends to be associated with *negative affect* when it is extrinsically motivated [31,33,34]; scholars have also demonstrated that both positive and negative affect predict psychological well-being, and each independently mediated the effect of trait emotional intelligence on life satisfaction [35].

Indeed, research consistently indicated a close association between PA and dimensions of affect (see Reed and Buck, 2009 and Reed and Ones 2006 for general overview). For example, PA appears to increase one’s ability to control negative affect through cognitive reappraisal [36]. More time spent doing moderate and vigorous physical activity (MVPA) is associated with higher positive affect and lower negative affect [37]. Overall, positive affect is negatively associated with the dimensions of mental illness/distress, whereas negative affect is positively associated with the indicators of PD [38]. The causal order of associations between PA and affect is not always clear [39,40,41]. Notably, positive affect and negative affect are not simply opposites. People can experience similar levels of both simultaneously [42,43,44]. It is possible that each exerts a certain degree of independent effects on the same psychological outcomes.

Research is scarce that accounts for positive and negative affect in the association between PA and psychological outcomes. Furthermore, attention to cultural context is often lacking in such a research focus. This study analyzed data from young adults in Iran. As representative studies show [45,46], insufficient physical activity (IPA) is rather prevalent in Iran. The latter study recorded a national average of 51.3% of people engaging in insufficient activity, citing additional risk factors such as urban residence, higher wealth, and being married. Cultural expectations surrounding gender can be assumed to contribute to women generally living a sedentary lifestyle, and this is reflected in empirical findings [45,46]. Perhaps physical activity among women in Iran contributes more to negative affect, putting women at risk for negative health outcomes. Providing psychological support and additional facilities for physical activity appears to have the potential to improve women’s physical activity and associated health [47]. Cross-cultural research showed that, while there was no difference between Iranian and Swedish participants in their general level of life satisfaction, differences existed regarding positive and negative affect [48]. Swedish participants reported relatively more positive affect than Iranian participants. Negative affect had the opposite pattern. In the Swedish sample, the most predictive factor of flourishing was positive affect, whereas a balance between positive and negative affect was more predictive of flourishing for the Iranian sample. These findings suggest that culture (or other macro influences) can contribute to the context in which affect plays a role in psychological processes. Given this background, the novelty of the present study consists of providing timely research on the associations between physical activity patterns and positive and negative affect in the Iranian cultural context.

### 1.2. The Current Study

The key purpose of the present research was to shed some more light on the complex and intertwined associations between physical activity patterns and psychological well- and ill-being, while considering affect as a possible mediator. More specifically, the current study aimed to explore the association between PA alongside dimensions of affect and psychological outcomes (PWB and PD) among Iranian young adults. Using structural equation modeling (SEM), we put particular emphasis on direct associations between PA with PWB and PD, and indirect associations between PA and PWB and PD as mediated by both positive and negative affect. This study has the potential to contribute to a better understanding of the mechanisms underlying the associations between PA and psychological outcomes.

The following six hypotheses guided the analyses. First, we expected that higher PA would be associated with higher PWB and lower PD (H1). Second, we anticipated that higher scores for positive affect would be associated with higher PWB and lower PD (H2). Third, we expected that higher scores for negative affect would be associated with lower scores of PWB and higher scores of PD (H3). Fourth, we expected that PWB indicators would be independently predicted by higher PA, higher positive affect, and lower negative affect (H4). Fifth, we predicted that PD indicators would be predicted independently by lower PA, lower positive affect, and higher negative affect (H5). Finally, we anticipated that positive and negative affect would at least partially mediate associations between PA and PWB and PD (H6).

## 2. Materials and Methods

### 2.1. Procedure

During the spring term of 2019, all second-semester students of the Faculty of Medicine of Kermanshah University (Kermanshah, Iran) were approached to participate in the study. Faculty members informed them about the study during classes. Inclusion criteria were: (a) at least 18 years old; (b) signed written informed consent; (c) students of different fields of the Kermanshah University of Medical Sciences; (d) and willing and able to complete the questionnaires. Participants were not reimbursed for their participation.

Eligible participants were informed about the aims of the study and the confidential and anonymous data handling and completed the booklet over a period of 20–30 min after the last session of the day. It included a series of questionnaires covering sociodemographic data, physical activity (PA), positive and negative affect, and various indicators of well-being and psychological dysfunctioning. The ethics committee of Kermanshah University of Medical Sciences (KUMS; Kermanshah, Iran; code: KUMS.REC.1395.304) approved the study, which was conducted in accordance with the rules laid down in the seventh and current edition of the Declaration of Helsinki.

### 2.2. Sample

In total, 213 students were approached, and 200 (86.6%) agreed to participate. All participants identified themselves as Iranian and healthy (physical and psychological). Participants were on average 24 years old (mean: 24.3; SD: 2.23). Roughly two thirds (n = 128; 64%) were female, and one third was male (n = 72; 36%). Most reported to be single (n = 164; 82%), whereas 36 participants (18%) reported to be married. The majority were Bachelor students (n = 138; 69%), although Master (n = 54; 27%) and PhD students (n = 8; 4%) were sampled too.

The topic of sample size and statistical power briefly needs to be addressed here; we later elaborate it further in the discussion section. At the time of data collection, no easily applicable tool was available regarding parallel mediations with latent variables. In line with an existing web application assuming manifest variables [49], we conservatively estimated that a sample size of 200 would be necessary. Recently, another web application [50], which also offers power-based sample size estimates for models with latent variables, suggested a sample size of 220, assuming a medium effect size [51], and previously established scale reliabilities or otherwise good reliability [52]. As we illustrate in Appendix B and readdress in the discussion section, ad hoc power analyses using the emergent model characteristics come to a different conclusion, highlighting certain sample size limitations of the current study.

### 2.3. Measures

#### 2.3.1. Psychological Well-Being Measures

***Life Satisfaction.*** Participants completed the Satisfaction With Life Scale administered in Farsi/Persian (Cronbach’s α = 0.83 [53]). The questionnaire consists of five items. Sample items are: “In most ways my life is close to my ideal,” and, “The conditions of my life are excellent.” Responses are given on seven-point scales ranging from 1 (strongly disagree) to 7 (strongly agree). Higher scale scores reflect a greater satisfaction with life.

***Physical Health.*** Participants completed the Physical Health subscale of the Iranian Lifestyle Questionnaire [54] as an indicator of well-being. This self-rating questionnaire consists of 8 items and focuses on overall physical health. Sample items are: “I try to keep my body healthy and bouncing,” “I regularly see a doctor for medical examinations,” and, “I have no chronic illnesses or physical disabilities.” Answers are given on 6-point rating scales ranging from 1 (absolutely disagree) to 6 (absolutely agree), with higher scale scores reflecting higher levels of physical health.

***Avoiding Drugs/Narcotics.*** To assess avoiding substance abuse as an indicator of well-being, we used the Avoiding Drugs and Narcotics subscale (ADNS). The ADNS is another subscale of the Iranian Lifestyle Questionnaire [54]. It consists of six items, and every item has a set of six possible responses. Sample items are: “I avoid drinking alcohol,” “I avoid arbitrary and unnecessary drug use,” and “I avoid associating with addicts and alcoholics.” Answers are given on 6-point rating scales ranging from 1 (absolutely disagree) to 6 (absolutely agree), with higher scale scores reflecting lower levels of substance and drugs abuse.

#### 2.3.2. Psychological Dysfunctioning Measures

To assess psychological dysfunctioning, participants completed the Farsi/Persian version of the General Health Questionnaire (GHQ-28) [55]. The GHQ is a self-rating questionnaire used to identify psychological distress. It consists of 28 items and assesses anxiety and insomnia, depression, social dysfunction, and somatic health. Answers are given with a four-point Likert scale ranging from 0 (not at all) to 3 (more than usual), with higher scores reflecting more severe health issues.

#### 2.3.3. Physical Activity

To assess physical activity, we used the Sport + Fitness subscale (SFS). The SFS is yet another subscale of the Iranian Lifestyle Questionnaire [54]. It consists of seven items, and every item has a set of six possible responses. Sample items are: “I exercise and strengthen my muscles at least a few times a week,” “I spend at least 30 min a day on vibrant physical activities such as fast hiking,” and, “I spend most of my spare time exercising or doing physical activity such as biking, hiking, swimming, and other sports.” Answers are given on 6-point rating scales ranging from 1 (absolutely disagree) to 6 (absolutely agree), with higher scale scores reflecting higher levels of physical activity.

#### 2.3.4. Dimensions of Affect

Participants completed the well-established psychometrically sound Farsi/Persian translation of the Positive and Negative Affect Schedule (PANAS-X) [56]. The PANAS-X is a self-rating scale, consisting of 60 items measuring positive and negative affect and focusing on *Basic Negative Emotion Scales* (fear, hostility, sadness, guilt), *Basic Positive Emotion Scales* (joviality, self-assurance, attentiveness), and *Other Affective States* (shyness, fatigue, serenity, surprise). Responses are given on a five-point rating scale ranging from 1 (very slightly or not at all) to 5 (extremely), with higher scale scores reflecting a more positive or more negative affect, respectively.

### 2.4. Statistical Analysis

Our statistical analyses, which were conducted to test our six hypotheses, made use of the so-called parceling approach [57]. Parceling means that, instead of working exclusively with single items (manifest variables) as indicators of a latent variable, we started by averaging scores across an appropriate number of items and then used these means as indicators of a latent variable. Parceling can be a useful analytical tool when a low subject-to-parameter ratio can be expected. This applies to the current study, given the use of the psychometrically established but multi-item scales that we presented in Section 2.3. The literature cautions against blindly calculating parceled scores, even when psychometrically established scales are involved. Instead, the literature warrants psychometric exploration of the involved multi-item scales before parceling can be applied [58]. Before calculating aggregate parceled scores from the psychometric scales, their dimensionality was thus explored via parallel analysis (within the software R; R Core Team, 2021) and exploratory factor analysis (EFA). In the latter, we followed the recommendations of Boateng and colleagues [59] to choose the extraction technique *Principal Axis Factoring,* and *Varimax* rotation. Further details are provided in the Appendix A. Table 1 (in the manuscript) shows that the most utilized instruments were uni-dimensional, and thus, parceling could be applied as-is. For the joviality, fear, and guilt subscales, one item each had to be excluded due to negative correlations with the other, semantically similar items, presumably due to misunderstandings of connotation (see the Appendix A for further details). For the subscale somatization, uni-dimensionality only emerged after excluding two items. Instead of the 7-item subscale of physical activity, a shorter 4-item version was preferred, since the explained variance and scale reliability largely improved. The Appendix A offers details on how the excluded items concern evaluative statements, whereas the remaining 4 items concern actual behavior. After corroborating uni-dimensionality for each subscale, mean index scores were computed in the manner suggested in the applicable scale manuals.

Utilizing these mean index scores, Table 2 depicts correlations between PWB indicators (life satisfaction, physical health, avoiding drugs and narcotics), PD indicators (depression, insomnia and anxiety, somatic health, social dysfunction), dimensions of affect (positive and negative affect), and physical activity. The predictive potential of physical activity, positive affect, and negative affect towards individual PWB and PD indicators was further explored in a set of regression analyses. In a following step, a confirmatory factor analysis (CFA) was conducted to test the parameters of expected (higher-order) latent variables (PA, dimensions of affect, PWB, and PD). Lastly, an SEM analysis was executed with PWB and PD as dependent variables, and dimensions of affect were seen alongside PA as predictors and dimensions of affect as mediators. The nominal level of statistical significance was set as α < 0.05. Additionally, bias-corrected percentile bootstrapping (10.000 iterations) at the same nominal level of statistical significance was employed to estimate the significance of direct and indirect effects. Statistical computations were performed with SPSS^®^ and AMOS^®^ 25.0 (IBM Corporation, Armonk, NY, USA) for Apple Mac^®^. Specific indirect effects were computed via MPlus v. 8.6 (Muthen and Muthen, 2017).

## 3. Results

### 3.1. Preliminary Analyses

#### 3.1.1. Associations between Study Variables

Table 2 reports descriptive statistics and correlation coefficients for PWB, PD, physical activity (PA), and dimensions of affect. Correlations are in line with H1, H2, and H3.

Regarding predictor variables (PA, positive and negative affect), higher physical activity (PA) and higher positive affect were associated with higher levels of all three PWB domains (satisfaction with life, physical health, and avoiding drugs and narcotics). Higher negative affect was associated with lower levels of all three PWB domains (see Table 2). Contrastingly, higher physical activity (PA) and positive affect were associated with lower levels of all four PD domains (somatization, anxiety and insomnia, depression, and social dysfunction). Higher negative affect was associated with higher levels of all four PD domains (*p* < 0.01). Finally, negative associations emerged between PWB and PD domains.

#### 3.1.2. Predicting PWB and PD from Physical Activity (PA) and Dimensions of Affect

Three hierarchical regression analyses were conducted to evaluate the influence of predictor variables (PA, positive affect, negative affect) on three domains of PWB (satisfaction with life, physical health, and avoiding drugs and narcotics). Results are displayed in Table 3 and are generally concordant with H4. PA predicted all PWB domains. Life satisfaction was predicted by negative and by positive affect. The latter also predicted the PWB domain avoiding drugs and narcotics. Interestingly, dimensions of affect were significant predictors of physical health when entered into the regression separately alongside PA but not when jointly included.

Four hierarchical regression analyses were conducted to evaluate the influence of predictor variables (PA, positive affect, negative affect) on four domains of psychological dysfunctioning (somatization, anxiety and insomnia, social dysfunction, depression). Physical activity, positive affect, and negative affect were significant predictors of the three first-mentioned domains of PD, whereas only positive and negative affect predicted depression (cf. Table 3). These results were concordant with H5.

### 3.2. Measurement Models

Several authors [60,61] suggested conducting a confirmatory factor analysis (CFA) to examine whether the measurement models provide an acceptable fit to the data. Once an acceptable measurement model is developed, the structural model can be examined [60]; an acceptable model should reach cut-off values close to 0.95 for the Comparative Fit Index (CFI), Normed Fit Index (NFI), Goodness-of-Fit Index (GFI), Incremental Fit Index (IFI), and Tucker-Lewis Index (TLI), in combination with cut-off values close to 0.08 for standardized root mean squared residual (SRMR) and root mean squared error of approximation (RMSEA) to evaluate model fit.

One of the statistical presuppositions necessary for the existence of a mediator relationship between variables is that the regression of the predictor variable on the mediator variable emerges as significant. As PA is the only predictor variable of the mediators in our models, Column 1 in Table 2 corroborates this.

The measurement model resulted in a good fit to the data (cf. Table 4). The loadings of the measured variables on the latent variables of PWB and PD and positive and negative affect were statistically significant at the 0.001 level. This implied that PWB, PD, positive affect, and negative affect appeared to have been adequately measured by their respective indicators. Furthermore, correlations between variables were statistically significant (cf. Table 4).

### 3.3. Structural Models

It was assumed at the outset of the study that PWB and PD would be the outcome variables, that physical activity (PA) would be the predictor variable, and that positive and negative affect would be potential mediator variables. We conducted SEM analyses to test a causal model between the predictor (and mediators) variables and two groups of dependent variables. Maximum likelihood was used as the estimation method.

### 3.4. Direct and Indirect Effects of PA on PWB, with Dimensions of Affect as Mediators

With the above considerations in mind, the PWB structural model was configured, with PA as the independent variable with direct and indirect paths to PWB, and with positive and negative affect as mediating variables. The latent variable of physical activity (PA) was established as the predictor variable, using as indicators its four items. Satisfaction with life (SWLS), physical health (PH), and avoiding drugs and narcotics (AND) were specified as indicators of the psychological well-being (PWB) outcome latent variable. Joviality (J), self-assurance (SA), and attentiveness (A) were treated as indicators of a latent mediator variable that we labeled as positive affect. Fear (F), guilt (G), hostility (H), and sadness (S) were treated as indicators of a latent mediator variable that we labeled as negative affect.

**Figure 1:**
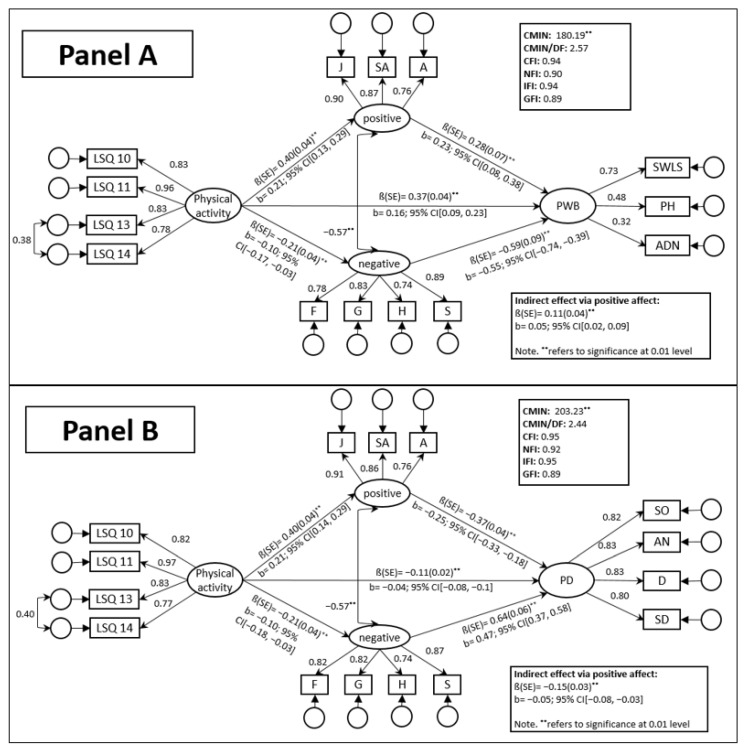
The structural models with PWB (panel **A**) and PD as outcome variables (panel **B**).

The model yielded a good fit to the data (cf. Figure 1 panel A). Although the χ^2^-value was statistically significant, the other indices were close to the recommended range. The paths from physical activity to PWB, positive affect, and negative affect were statistically significant, as were the paths from positive and negative affect to PWB. Additionally, physical activity had a significant indirect effect on PWB via positive affect and also via negative affect (cf. Figure 1 panel A).

In summary, taken in isolation, greater levels of physical activity (PA) predict higher levels of psychological well-being (PWB). However, the dynamic becomes more complex when positive affect and negative affect are taken into account. It appears that the effect of physical activity (PA) on psychological well-being (PWB) is partially mediated by positive affect. Specifically, higher levels of physical activity (PA) were associated with higher levels of positive affect, which in turn were associated with greater levels of psychological well-being (PWB). Negative affect seems to likewise partially mediate the association between physical activity (PA) and psychological well-being (PWB), albeit in the opposite direction.

### 3.5. Direct and Indirect Effects of PA on PD, with Dimensions of Affect as Mediators

The same analytic procedures were used for the model predicting PD. The latent variable of physical activity (PA) as well as positive and negative affect were estimated the same way as in the model predicting PWB. The current model differed from that model in that it had psychological dysfunction as the outcome latent variable (specified via the indicators somatization (S), anxiety and insomnia (A), depression (D), and social dysfunction (SD).

As with the model for PWB, the model fit indices were good. Although the χ^2^-value was statistically significant, the other indices were close to the recommended range. The paths from physical activity to PD, positive affect, and negative affect were statistically significant, as were the paths from positive and negative affect to PD. Additionally, physical activity had a significant indirect effect on PD via positive affect and also via negative affect (cf. Figure 1 panel B).

In summary, taken in isolation, greater levels of physical activity (PA) predicted lower levels of psychological dysfunction (PD). However, the dynamic became more complex when negative affect and positive affect were taken into account. In this latter case, it appears that the effect of physical activity (PA) on psychological dysfunction (PD) was partially mediated by their positive affect indicators. Specifically, higher levels of physical activity (PA) were associated with higher levels of positive affect, which in turn were associated with lower levels of psychological dysfunction (PD). Again, negative affect appeared as another different mediator in the relationship between physical activity (PA) and psychological dysfunctioning (PD), albeit in opposite direction.

Taken together, the results from both models aligned with H6. Positive and negative affect emerged as significant (partial) mediators in the relationship between physical activity and PWB, as well as the relationship between physical activity and PD. Accordingly, change in affect might be one mechanism underlying the association between physical activity and (un)favorable psychological functioning.

## 4. Discussion

The findings at hand indicate that, among a sample of young Iranian adults, physical activity (PA) and dimensions of affect were associated with favorable (PWB) and unfavorable (PD) psychological functioning. Furthermore, PA and dimensions of affect were independent and significant predictors of indicators of PWB (i.e., life satisfaction, physical health, avoiding drugs and narcotics) and indicators of psychological dysfunction (PD) (i.e., somatic symptoms, anxiety and insomnia, social dysfunction, and depression). Most importantly, the results of SEM analyses showed that positive and negative affect play a mediating role between physical activity and PWB and PD. This indicates that change in affect might in part explain the frequently observed association between physical activity and (un)favorable psychological functioning, also in young adults from a non-western cultural background.

Six hypotheses were formulated and are discussed in the following. As expected in H1, higher PA was associated with higher PWB and lower PD among young Iranian adults, confirming what past research found regarding other samples [1,2,3,12,15,30,62,63]. Consequently, we believe that interventions to promote PA in Iran and elsewhere would likely have a positive impact on indicators of PWB and reduce PD. Furthermore, the results suggest that higher PA scores appeared to impact both positive and negative psychosocial dimensions. Since both are not necessarily co-occurring (cf. Table 2), higher PA levels appeared to be associated with a “double” psychological benefit.

As expected in H2, higher scores of positive affect were associated with higher PWB and lower PD for the Iranian sample, consistent with previous findings for other samples [17,37,41,64,65]. More specifically, we confirmed that the previous finding of a relationship between positive affect and higher PWB or lower PD also applies in early adulthood in a non-Western context. Similarly, and confirming our third hypothesis, similar to other studies [41,66,67,68,69,70], higher scores of negative affect were associated with lower PWB and higher PD.

To explain the results of H2 and H3, the present data do not provide any deeper insight into underlying psychological or physiological mechanisms, though previous studies showed that dimensions of affect might be an outcome of regular PA [39,71,72] or might result in PA [41,73,74,75]. Based on prior research, we anticipated that positive affect and negative affect would be relevant to PA and PWB/PD and are worth investigating. Yet, ultimately, experimental studies are necessary to assess causality.

In general agreement with our fourth hypothesis, PWB indicators (life satisfaction, physical health, and avoiding drugs) were independently predicted by high PA, high positive affect, and low negative affect. The results were rather clear-cut (see Table 3). However, some variations emerged per indicator, e.g., the relative contribution of negative and positive affect did not reach statistical significance for physical health, although *p* values were below 0.10 and only rose above 0.05 when entering both dimensions of affect jointly into the equation. As per direct health consequences, it could be that PA alone plays a more important role in physical health than affect (or a reverse causal link inflates such a connection). Similarly, PA was the strongest predictor for avoiding drugs/narcotics, and the relative contribution of positive affect did not reach significance. Yet, per the cultural setting, this could differ in contexts with less restrictive laws on substance use. Negative affect emerged as the strongest predictor for life satisfaction. The latter resonates with the idea of a negativity bias regarding life satisfaction [76]: negative affect seemingly often does more harm to life satisfaction than positive affect does to enhance it.

In our fifth hypothesis, we expected that PD indicators (somatic symptoms, anxiety and insomnia, social dysfunction, and depression) would be predicted independently by lower PA, poorer positive affect, and higher negative affect. The results were rather clear-cut (see Table 3). Specifically, PA in combination with dimensions of affect explained 55%, 58%, 55%, and 59% of the variance in models of somatic symptoms, anxiety/insomnia, social dysfunction, and depression, respectively. However, physical activity was no longer a significant predictor of depression when also considering dimensions of affect. Given their high ß coefficients, we interpreted that, for this subdimension of PD, a full mediation via dimensions of affect are viable. Negative affect was the strongest predictor on all subdimensions, except for social dysfunction, where positive affect was the strongest predictor. Our results are generally consistent with research that found connections between affect and somatic symptoms [77,78,79,80], depression [81,82,83,84,85], anxiety [86], and social dysfunction [38,87]. Our results also indicate that affect predicts such outcomes above and beyond PA, at least for Iranian young adults.

The above-mentioned regression results generally supported H4 and H5. The measurement model showed that the specific subdimensions of PWB and PD could indeed be grouped into those higher order constructs (cf. Table 4). Yet, the regression results highlight that some variability does exist concerning the relative contribution of PA, positive affect, and negative affect to the specific PWB and PD subdimensions. Although beyond the scope of the current study, further exploring this and the mediating potential of affect per subdimension could be an avenue for future research.

We found support for our sixth hypothesis, that affect would mediate the relationship between physical activity and favorable and unfavorable psychological functions. Namely, PA was related to positive and negative affect in the structural equation models. PA directly predicted PWB and PD and indirectly predicted PWB and PD via positive and negative affect.

Results of the present study, informed by a health psychology framework, provide support for one apparent dynamic underlying the PWB and PD phenomenon. In isolation, it appears that Iranian adults with higher levels of PA tend to exhibit greater levels of PWB and lesser levels of PD. Presumably, higher levels of physical activity may increasingly sensitize Iranian young adults to the need to modify how they experience psychological functioning in the inside/outside world. However, this isolated effect appears to belie a more complex dynamic. Using the present data, we were able to account for the additional factors of positive and negative affect. Results were consistent with the premise that greater levels of physical activity lead to a stronger positive affect (and lower negative affect), which in turn produces higher levels of PWB and lower levels of PD. As such, it would appear that efforts to link dimensions of affect to PA and enhance levels of positive affect (and decrease levels of negative affect) during PA could be an effective means toward boosting psychological functioning. Additionally, positive affect (and the absence of negative affect) likely reinforces the desire to engage in PA, thus making positive affect an important key in promoting and sustaining health behaviors and outlooks.

Despite the novelty of findings, several limitations warrant against the overgeneralization of results. First, we relied entirely on self-report data. More objective measures of PA could yield different results. Second, the present pattern of results might be due to further latent, unassessed dimensions, which could have biased two or more variables in the same direction. This holds particularly true for anthropometric data (height; weight; BMI). Third, no objective physiological data were collected; such data might have allowed us to illuminate the underlying neurophysiological processes linking physical activity, dimensions of affect, and PWB (or PD). Fourth, the data do not provide insights into possible work-related, stress-related, or motivational issues underlying current PWB, PD, PA, and dimensions of affect. Though highly speculative, one might assume that workload, job insecurity, family strain, financial issues, academic stresses, or further, e.g., sociopolitical stressors, might have an impact on cognitive emotional processes involved in PWB and PD indicators and in PA. Thus, under certain circumstances, PA could be especially effective, producing positive affect and promoting positive psychological functioning, but the data did not include sufficient information to account for such circumstances. Fifth, given the close association of positive and negative affect with PD indicators, it is conceivable that PD indicators were an epiphenomenon of broader dimensions of affect. Sixth, a longitudinal or experimental study design would be needed to assess causality. Seventh, and relating to the previous point, the cross-sectional nature of the current study means that less distinction can be given to the conceptually important difference between trait- and state-related positive and negative affect. In a cross-sectional design, even a measurement that makes reference to affective states is simply a recollection of the participants overall estimation at the time of filling in the survey. Ideally, future research within longitudinal or experimental designs should assess affective states during or immediately after physical activity. Including additional affect-related items or introductory statements that make a reference to the trait-state distinction might also help. Eighth, and likewise relating to the study design, Appendix B highlights sample size-related limitations pertaining to statistical power. The method for conducting SEM power analyses described in [50] might guide future research to help minimize such issues, and model characteristics emergent from the current study might supply future research with parameters for conducting a priori power analyses to gauge sample size requirements.

## 5. Conclusions

The current research examined the associations between physical activity (PA), positive/negative affect, psychological well-being (PWB), or psychological dysfunctioning (PD) in an Iranian sample. The results indicate that PA and positive/negative affect are associated with PWB/PD. By evaluating causal models between variables, the current research expands the PWB and PD literature in non-Western cultures, more generally, and adds the literature concerning the associations between PA and affect dimensions and PWB/PD, specifically.

## Figures and Tables

**Table 1:** Dimensionality and factor structure behind the scales measuring study variables.

	Full Scale					Utilized Scale (if Items Were Excluded)				
	KMO *^a^*	Dimensionality *^b^* (Parallel Analysis)	Variance Explained	n	α	KMO *^a^*	Dimensionality *^b^* (Parallel Analysis)	Variance Explained	n	α
1. Physical activity	0.84 *	1	55%	7	0.90	0.81 *	1	74%	4	0.92
2. Positive affect	0.73 *	1	69%	3	0.87					
Attentiveness	0.76 *	1	56%	4	0.83					
Joviality	0.91 *	1	56%	8	0.80	0.92 *	1	61%	7	0.91
Self-assurance	0.89 *	1	57%	6	0.89					
3. Negative affect	0.83 *	1	66%	4	0.88					
Fear	0.79 *	1	43%	6	0.65	0.79 *	1	49%	5	0.81
Guilt	0.82 *	1	51%	6	0.84	0.81 *	1	59%	5	0.88
Hostility	0.82 *	1	51%	6	0.85					
Sadness	0.83 *	1	58%	6	0.87					
4. Somatization	0.74 *	2	29% 25%	7	0.78	0.80 *	1	43%	5	0.78
5. Anxiety	0.79 *	1	44%	7	0.84					
6. Depression	0.88 *	1	62%	7	0.92					
7. Social dysfunction	0.81 *	1	43%	7	0.83					
8. Life satisfaction	0.86 *	1	58%	5	0.87					
9. Physical Health	0.68 *	2	29% 8%	8	0.59		1		2	0.92
10. Avoiding Narcotics	0.74 *	1	57%	6	0.88					

Note. KMO = Kaiser-Meyer-Olkin test. *^a^* * refers to a significant (*p* < 0.05) Bartlett’s test of sphericity. *^b^* for further details and scree plots, see the Appendix A.

**Table 2:** Descriptive statistics and bivariate correlations between study variables.

Variable	1	2	3	4	5	6	7	8	9	10
1. Physical activity	—									
2. Positive affect	0.38 **	—								
3. Negative affect	−0.15 *	−0.50 **	—							
4. Somatization	−0.44 *	−0.63 **	0.59 **	—						
5. Anxiety and insomnia	−0.30 **	−0.60 **	0.70 **	0.71 **	—					
6. Depression	−0.19 *	−0.57 **	0.74 **	0.68 **	0.66 **	—				
7. Social dysfunction	−0.35 **	−0.65 **	0.61 **	0.68 **	0.66 **	0.66 **	—			
8. Life satisfaction	0.38 **	0.55 **	−0.55 **	−0.56 **	−0.52 **	−0.59 **	−0.57 **	—		
9. Physical health	0.44 **	0.33 **	−0.24 **	−0.43 **	−0.40 **	−0.23 *	−0.21 **	0.29 **	—	
10. Avoiding narcotics	0.25 **	0.17 *	−0.22 *	−0.29 **	−0.24 **	−0.21 **	−0.13	0.26 **	0.35 **	—
Range	1–6	1–5	1–5	0–3	0–3	0–3	0–3	1–5	1–6	1–6
Mean	3.37	3.20	2.14	1.05	1.06	0.58	1.04	3.24	5.01	5.22
Standard deviation	1.51	0.69	0.71	0.58	0.57	0.63	0.46	0.80	0.89	1.01

Note. ** *p* < 0.01; * *p* < 0.05.

**Table 3:** Regression analyses of PA, and dimensions of affect predicting specific PD and PWB domains.

Model	Variable	Predictors *^a^*			Model Statistics
		Physical Activity	Positive Affect	Negative Affect	
PWB	Life satisfaction	0.22 ** [0.06 0.17]	0.28 ** [0.17 0.48]	−0.37 ** [−0.54 −0.29]	R^2^ = 0.44 F (3.196) = 50.919 **
	Physical health	0.37 ** [0.14 0.30]	0.13 [−0.02 0.33]	−0.13 [−0.36 0.02]	R^2^ = 0.23 F (3.196) = 19.980 **
	Avoiding narcotics	0.22 ** [0.04 0.25]	0.01 [−0.21 0.21]	−0.18 ** [−0.50 −0.04]	R^2^ = 0.09 F (3.196) = 6.718 **
PD	Somatization	−0.26 ** [−0.14 −0.06]	−0.34 ** [−0.38 −0.20]	0.38 ** [0.22 0.40]	R^2^ = 0.55 F (3.196) = 80.043 **
	Anxiety and insomnia	−0.11 ** [−0.08 −0.01]	−0.29 ** [−0.33 −0.15]	0.53 ** [0.34 0.51]	R^2^ = 0.58 F (3.196) = 90.659 **
	Social dysfunction	−0.14 * [−0.08 −0.01]	−0.41 ** [−0.35 −0.20]	0.38 ** [0.16 0.33]	R^2^ = 0.55 F (3.196) = 79.636 **
	Depression	0.01 [0.04 0.05]	−0.27 ** [−0.36 −0.14]	0.60 ** [0.43 0.62]	R^2^ = 0.59 F (3.196) = 96.779 **

Note. ** *p* < 0.01; * *p* < 0.05. *^a^* shown are ß-coefficients but brackets contain 95% confidence intervals of b-coefficients.

**Table 4:** Correlations among variables in the measurement model.

Latent Variable	1	2	3	4	5
1. Physical activity	—				
2. Positive affect	0.40 **	—			
3. Negative affect	−0.21 **	−0.59 **	—		
4. Psychological well-being (PWB)	0.61 **	0.78 **	−0.82 **	—	
5. Psychological dysfunction (PD)	−0.39 **	−0.78 **	0.88 **	−0.90 **	—

Note. ** *p* < 0.01; CMIN/DF = 2.55; CFI = 0.93; NFI = 0.88; GFI = 0.85; IFI = 0.93; TLI = 0.91; RMSEA = 0.09; SRMR = 0.06.

**Figure A1:**
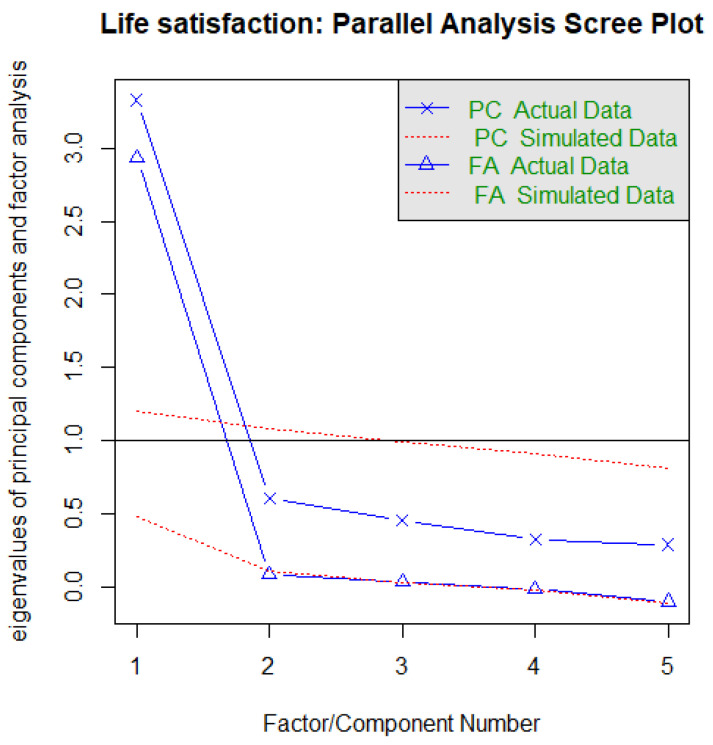
Results from the parallel analysis of the satisfaction with life scale (SWLS).

**Figure A2:**
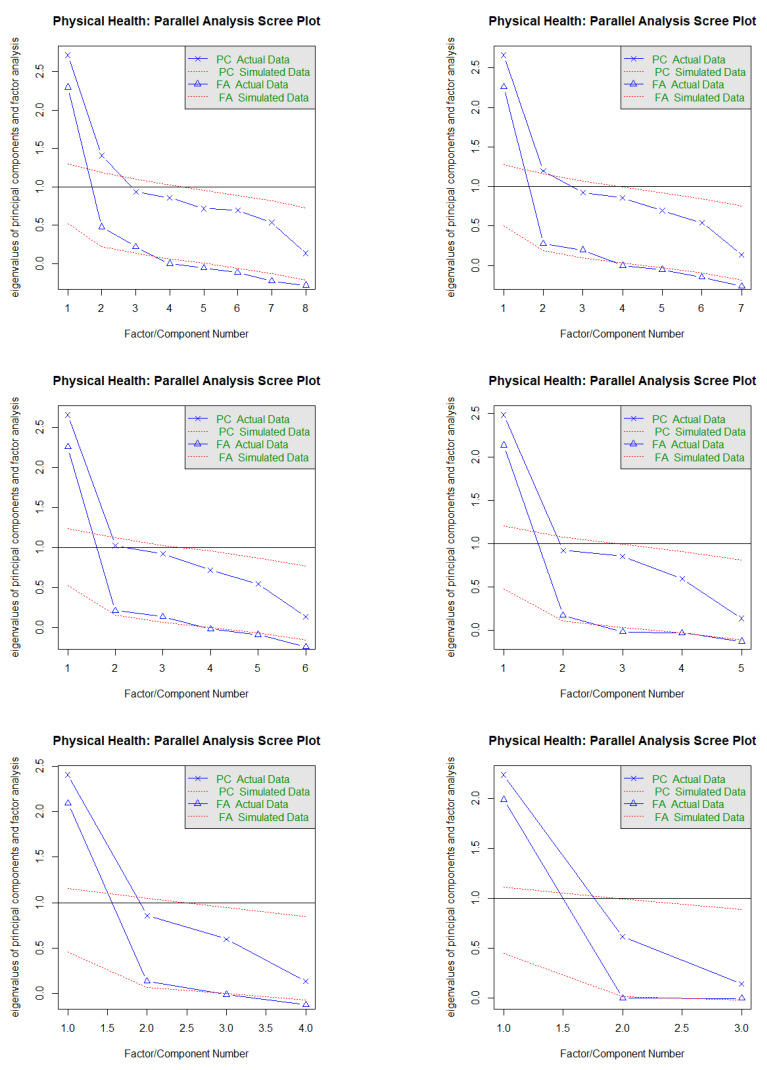
Results from the parallel analysis of the LSQ subscale physical health.

**Figure A3:**
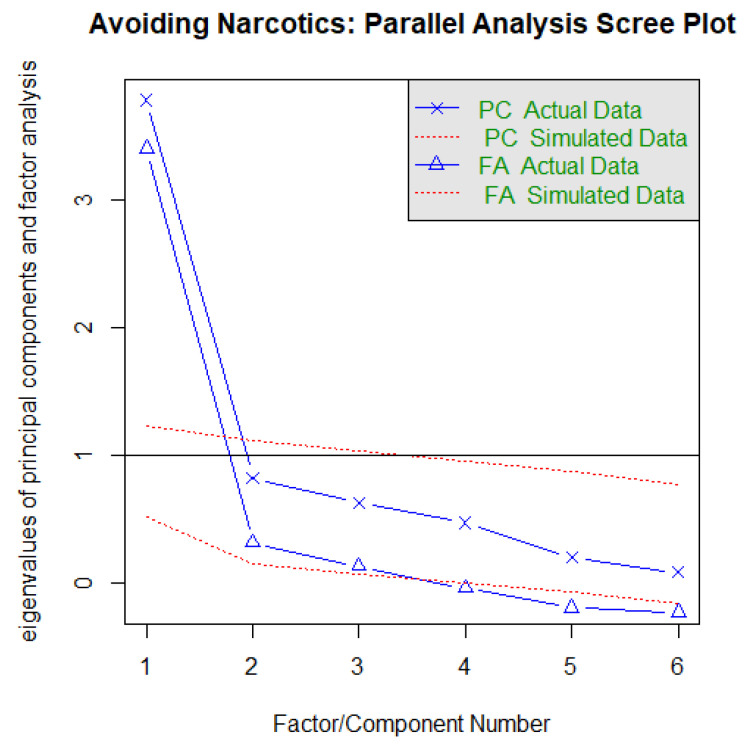
Results from the parallel analysis of the LSQ subscale avoiding drugs/narcotics.

**Figure A4:**
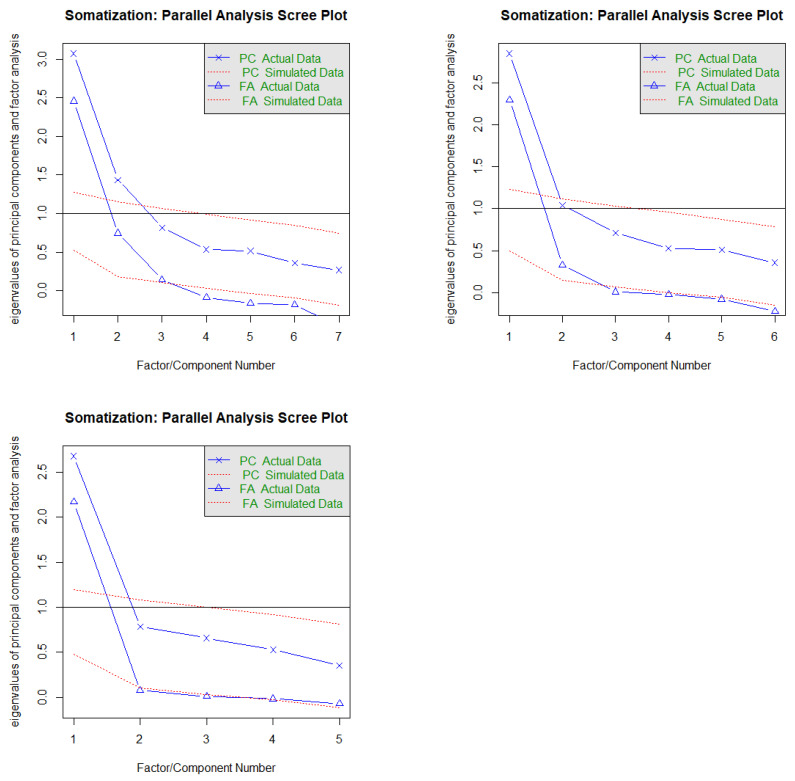
Results from the parallel analysis of the GHQ-28 subscale somatization.

**Figure A5:**
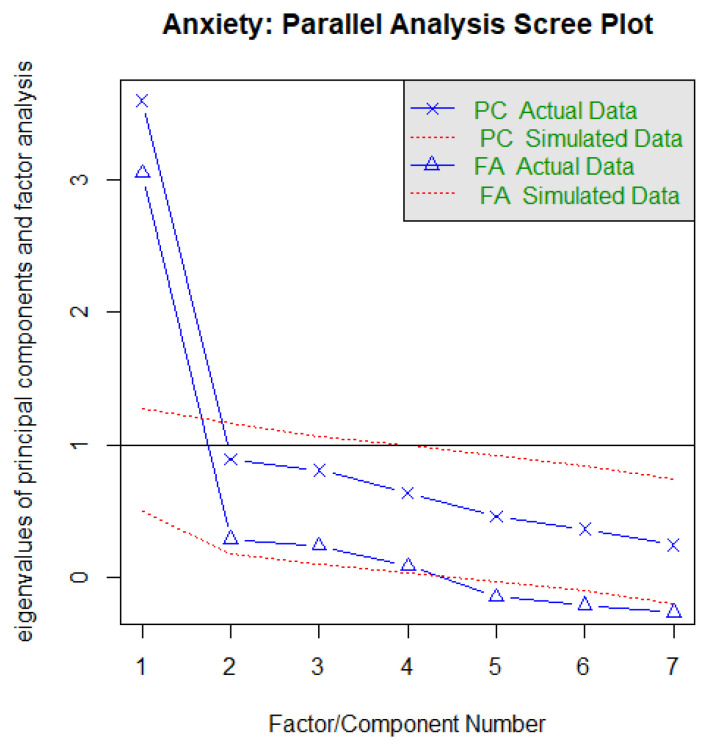
Results from the parallel analysis of the GHQ-28 subscale anxiety.

**Figure A6:**
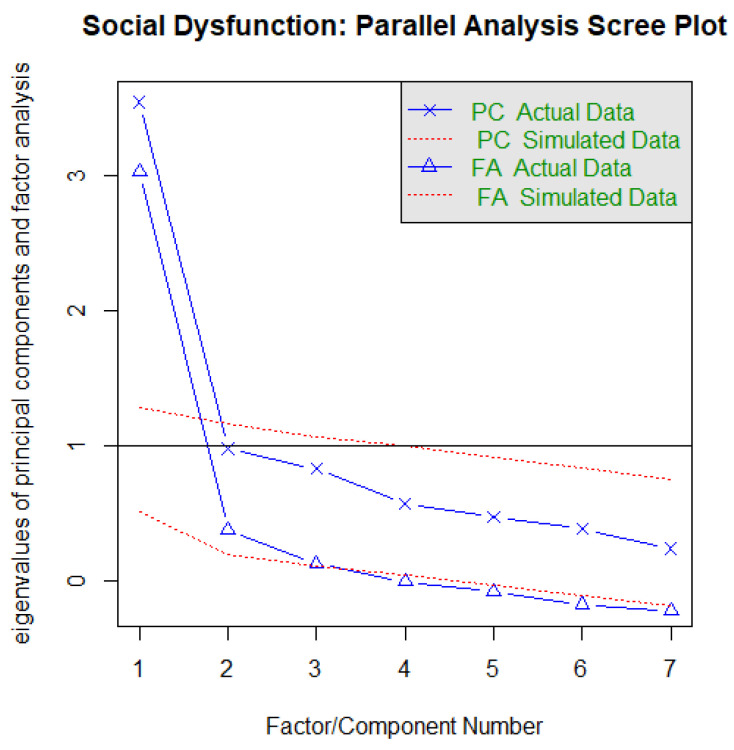
Results from the parallel analysis of the GHQ-28 subscale social dysfunction.

**Figure A7:**
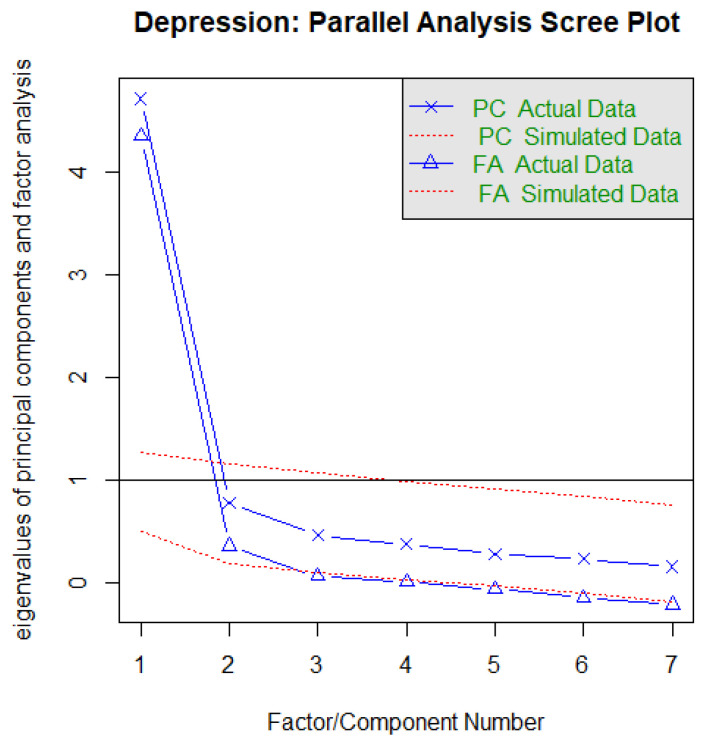
Results from the parallel analysis of the GHQ-28 subscale depression.

**Figure A8:**
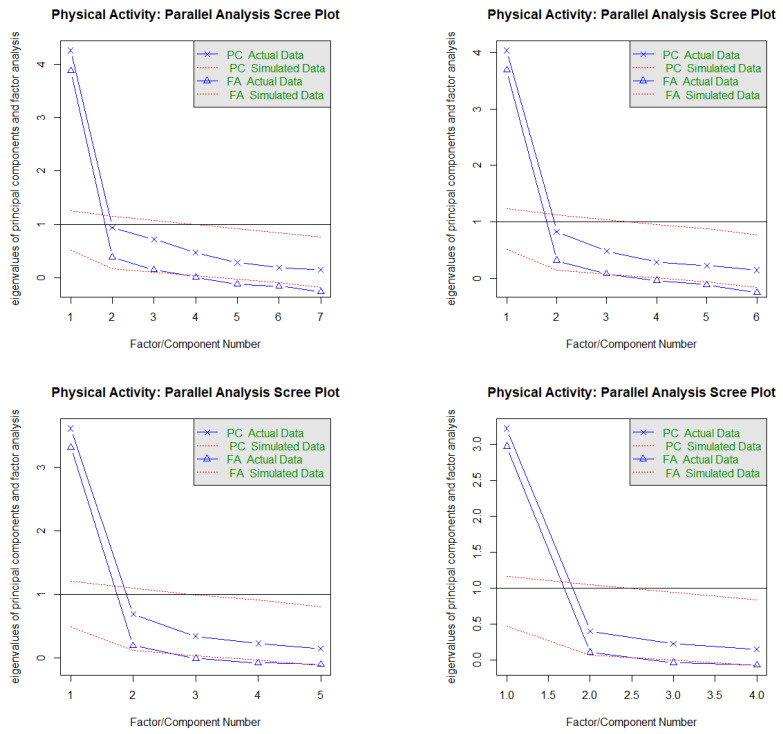
Results from the parallel analysis of the LSQ subscale physical activity.

**Figure A9:**
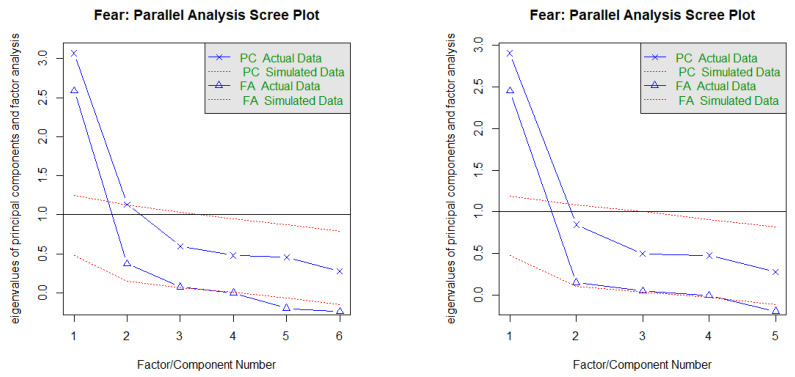
Results from the parallel analysis of the PANAS-X subscale fear.

**Figure A10:**
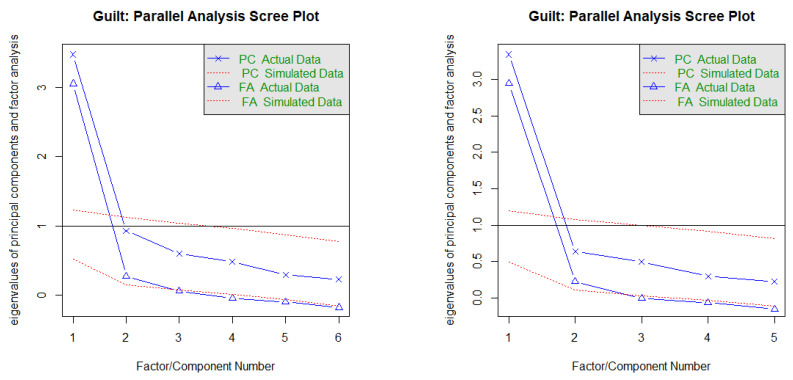
Results from the parallel analysis of the PANAS-X subscale guilt.

**Figure A11:**
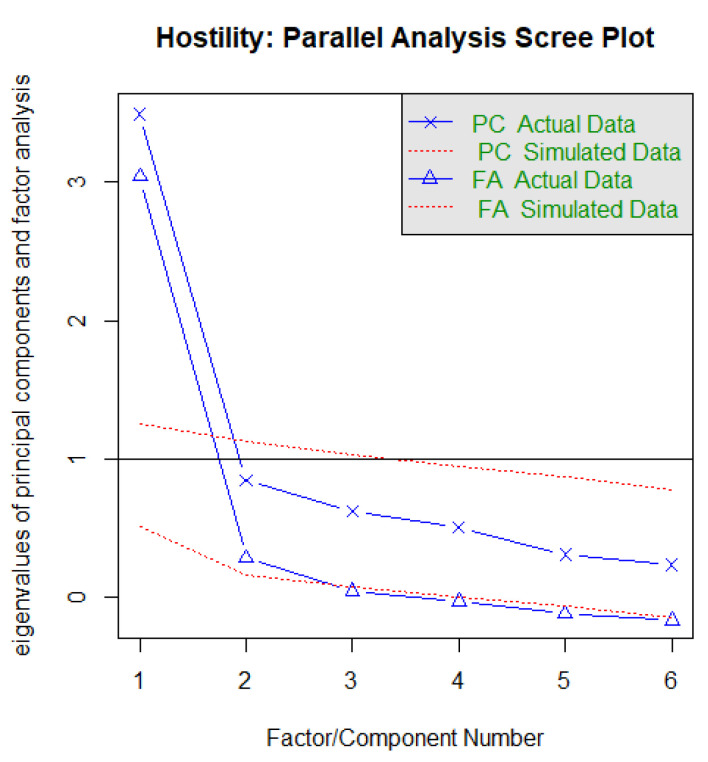
Results from the parallel analysis of the PANAS-X subscale hostility.

**Figure A12:**
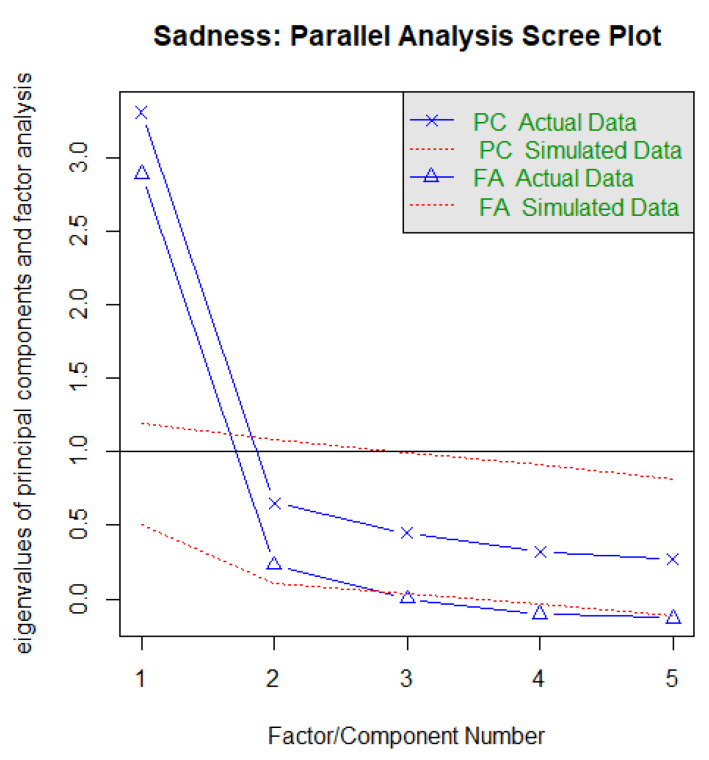
Results from the parallel analysis of the PANAS-X subscale sadness.

**Figure A13:**
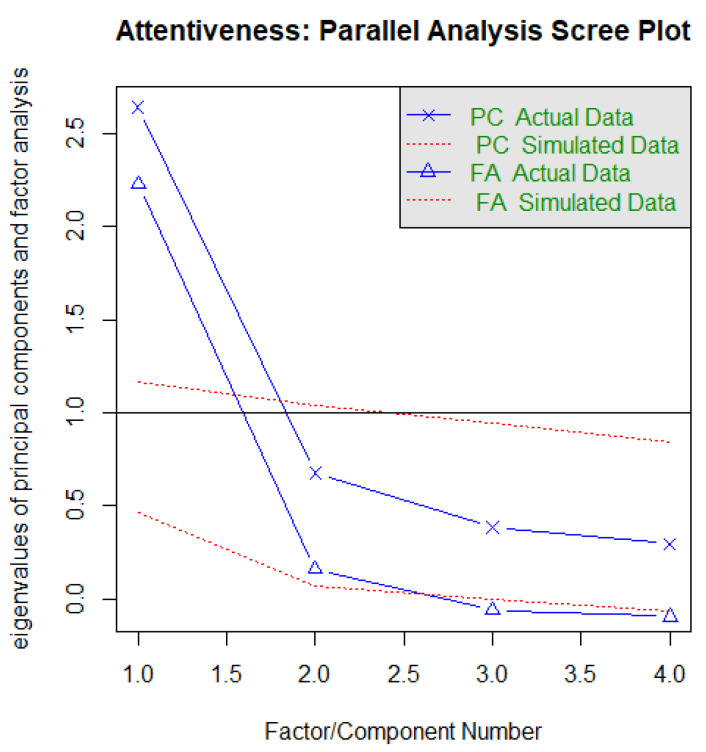
Results from the parallel analysis of the PANAS-X subscale attentiveness.

**Figure A14:**
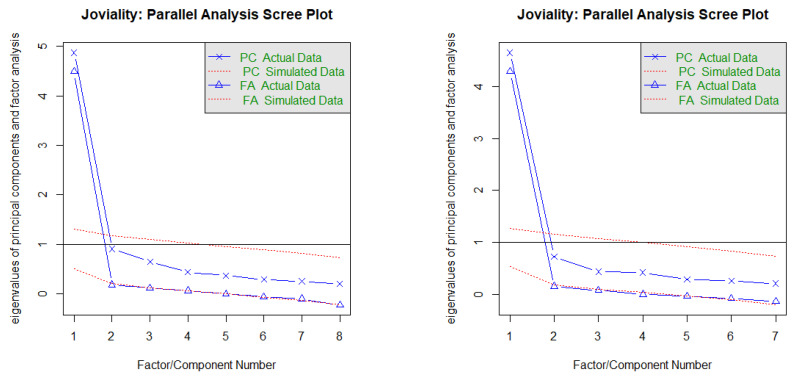
Results from the parallel analysis of the PANAS-X subscale joviality.

**Figure A15:**
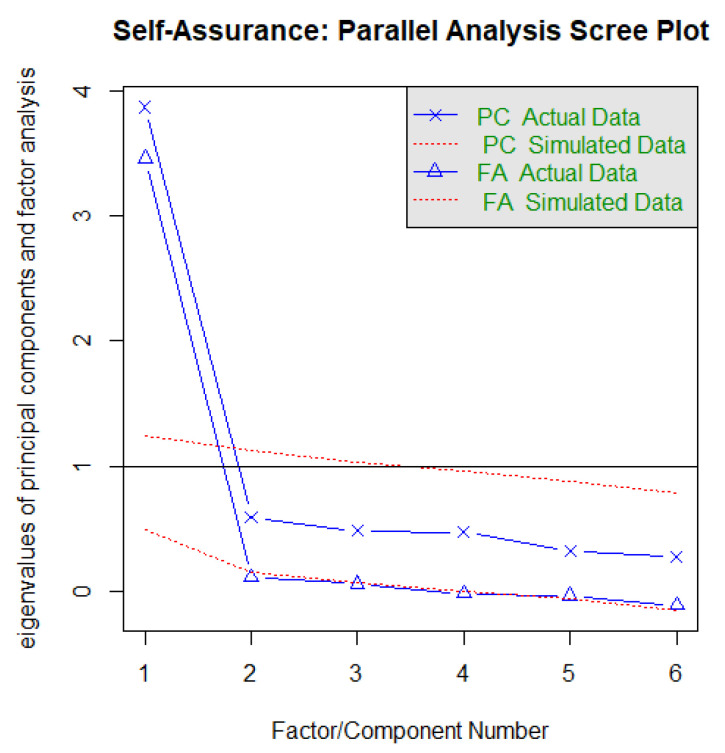
Results from the parallel analysis of the PANAS-X subscale self-assurance.

**Figure A16:**
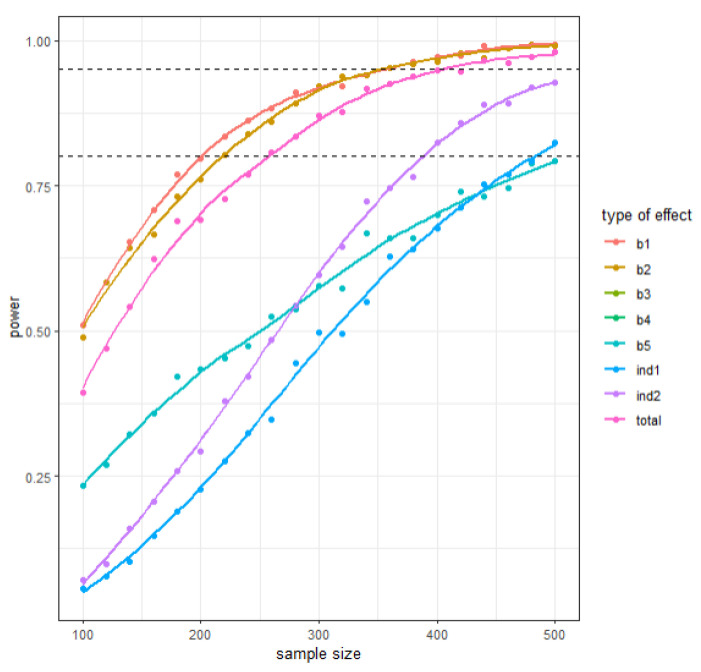
Power curve relating to the SEM model shown in panel A) of Figure 1.

**Figure A17:**
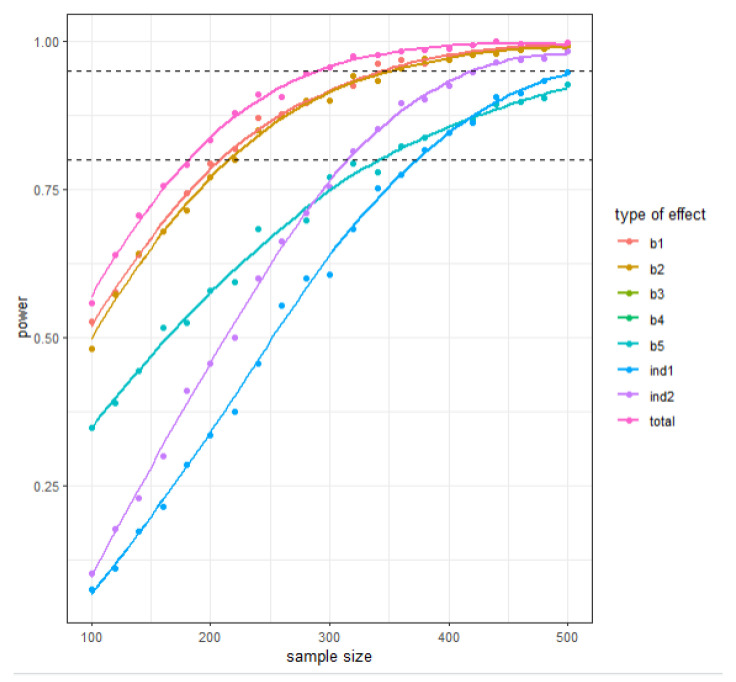
Power curve relating to the SEM model shown in panel B of Figure 1.

## Data Availability

The data that support the findings of this study are available upon request from the co-author, Nils T. Henschel. The data are not publicly available due to containing information that could compromise the privacy of research participants.

## Appendix A. Details and Dimensionality of Psychometric Scales

This appendix contains additional information on the utilized psychometric scales. In the first step, we explored their dimensionality. This step is generally recommended [59], especially when applying pre-existing scales in a novel context like we do. To this end, we conducted exploratory factor analysis (EFA) per scale, whereby we followed the analytical recommendations given in Boateng et al. [59]. Accordingly, we chose the extraction technique *Principal Axis Factoring* due to deviations from multivariate normality (cf. Table A1) and *Varimax* rotation, since we expected to extract one factor per construct-specific item pool. Initial factorability was assessed with the Kaiser-Meyer-Olkin measure and Bartlett’s test of sphericity. The number of extracted factors was established by combinedly assessing Kaiser’s Eigenvalue criterion, scree plots, and parallel analysis (100 iterations via the function *fa.parallel* from the R package *psych*). Items with factor loadings below 0.40 were excluded iteratively, starting with the lowest. In this, cross-loadings and α-if-item-deleted values from reliability analysis were also taken into account. This procedure was repeated iteratively until a satisfactory solution emerged. The emergent scale’s reliability was interpreted as good if Cronbach’s α was >0.80 and as *acceptable* for values in the 0.70 s.

Some authors recommend to further assess dimensionality with model fit indices from confirmatory factor analysis ([59], Table 2). We refrained from this step firstly because some of these fit measures proved inaccurate in small samples with non-normal data like ours (cf. [59], Table 2). Secondly, emergent research indicates more contextual distortion—already from few, small residual correlations—and that simply following cut-off values can result in dimensional over-fitting. These authors recommend parallel analysis to establish dimensionality, which we conducted within our EFAs.

**Table A1:** Distributional properties and missing cases per construct (imputed by item mean).

Construct	Missing Items (Replaced by Mean)	Mardia’s Skewness	Mardia’s Kurtosis
Satisfaction with life	*SWLS1* (4), *SLWS2* (4), *SLWS3* (4), *SLWS4* (4), *SWLS5* (4)	3.396 *	39.509 *
Physical health	*LSQ3* (1), *LSQ4* (1), *LSQ5* (2), *LSQ6* (1), *LSQ7* (3), *LSQ8* (3)	25.177 *	108.232 *
Avoiding drugs/narcotics	*LSQ50* (6), *LSQ51* (6), *LSQ52* (6), *LSQ53* (6), *LSQ54* (6), *LSQ55* (6)	38.264 *	138.628 *
Somatization	*GHQ1* (1), *GHQ2* (1), *GHQ3* (1), *GHQ4* (1), *GHQ5* (1), *GHQ6* (3), *GHQ7* (1)	15.751 *	90.413 *
Anxiety	*GHQ8* (1), *GHQ9* (1), *GHQ10* (1), *GHQ11* (1), *GHQ12* (1), *GHQ13* (1), *GHQ14* (4)	8.318 *	75.527 *
Social dysfunction	*GHQ15* (4), *GHQ16* (4), *GHQ17* (6), *GHQ18* (9), *GHQ19* (6), *GHQ20* (4), *GHQ21* (5)	9.437 *	90.625 *
Depression	*GHQ22* (4), *GHQ23* (5), *GHQ24* (4), *GHQ25* (4), *GHQ26* (6), *GHQ27* (5), *GHQ28* (4)	19.385 *	95.055 *
Physical activity	*LSQ9* (3), *LSQ10* (2), *LSQ12* (3), *LSQ13* (6)	8.695 *	78.642 *
Fear	*afraid* (3), *scared* (4), *frightened* (2), *nervous* (3), *jittery* (4), *shaky* (2)	10.719 *	65.137 *
Guilt	*guilty* (3), *ashamed* (4), *blameworthy* (2), *angry at self* (2), *disgusted with self* (7), *dissatisfied with self* (2)	8.296 *	59.636 *
Hostility	*angry* (2), *hostile* (3), *irritable* (2), *scornful* (2), *disgusted* (2), *loathing* (2)	10.719 *	64.070 *
Sadness	*sad* (2), *blue* (3), *downhearted* (2), *alone* (2), *lonely* (2)	3.695 *	44.130 *
Attentiveness	*alert* (2), *attentive* (3), *concentrating* (2), *determined* (3)	1.059 *	28.774 *
Joviality	*happy* (6), *joyful* (5), *delighted* (6), *cheerful* (2), *excited* (5), *enthusiastic* (3), *lively* (5), *energetic* (2)	11.897 *	113.673 *
Self-assurance	*proud* (3), *strong* (3), *confident* (3), *bold* (4), *daring* (2), *fearless* (3)	11.169 *	73.747 *

Note. *N* = 200. The last two columns indicate violations of the multivariate normality assumption per construct-specific subscale, whereby significant values (*p* < 0.05) are marked with an asterisk.

### Appendix A.1. Life Satisfaction (PWB Indicator)

Exploratory factor analyses indicated good factorability (KMO = 0.86) with a one-factorial structure accounting for 58% of the variance. Factor loadings were above the 0.400 threshold. The reliability was satisfactory (α = 0.87).

**Table A2:** Results from the factor analysis of the satisfaction with life scale (SWLS).

Item	Factor Loading
1. In most ways, my life is close to the ideal.	**0.814**
2. The conditions of my life are excellent.	**0.775**
3. I am satisfied with life.	**0.848**
4. So far I have gotten the important things I want in life.	**0.762**
5. If I could live my life over, I would almost change nothing.	**0.612**

Note. *N* = 200. The extraction method was principal axis factoring with an orthogonal (varimax) rotation. Factor loadings above 0.40 are in bold.

### Appendix A.2. Physical Health (PWB Indicator)

Exploratory factor analyses indicated just mediocre factorability (KMO = 0.68) and low reliability (α= 0.59) of the set of eight items that were supposed to measure physical health. Based on the Eigenvalue criterion, two factors would be extracted. Under successive exclusion of the items that loaded highest on factor two but lowest on factor one, an internally consistent (α = 0.92) one-factorial three-item scale emerged (KMO = 0.81), which accounted for 74% of the variance and consisted of the items LSQ1, LSQ2, and LSQ4. For reasons of parameter parsimony, it was decided to form a mean score of items LSQ1 and LSQ2 as a measure of physical health. The resulting item (termed PH) correlated near perfectly with a factor score based on the three-item scale (r = 0.999 ***). We suspect that the original eight-item scale did not perform as expected in this sample of medical students for two reasons: Firstly, they might have a different understanding of the concept of distinguishing between chronic health aspects (LSQ5) and malleable aspects (the rest). Secondly, some items might not apply given their occupational training (LSQ7, LSQ3), the high workload that it entails (LSQ6), and their increased medical knowledge, which makes general comparisons within an age group (LSQ8) appear arbitrary.

**Table A3:** Results from the factor analysis of the LSQ subscale physical health.

Item	Factor Loadings (EFA 1)		Factor Loadings (EFA 2)		Factor Loadings (EFA 3)		Factor Loadings (EFA 4)	Factor Loadings (EFA 5)	Factor Loadings (EFA 6)
	Factor 1	Factor 2	Factor 1	Factor 2	Factor 1	Factor 2	Factor 1	Factor 1	Factor 1
1. LSQ1: I try to keep my body healthy and bouncing.	**0.876**	0.116	**0.872**	−0.005	**0.790**	0.353	**0.874**	**0.886**	**0.924**
2. LSQ2: I take care of my health.	**0.872**	0.219	**0.907**	−0.139	**0.902**	0.315	**0.941**	**0.942**	**0.929**
3. LSQ4: I am able to rest and relax.	**0.570**	0.203	**0.594**	−0.032	**0.454**	0.391	**0.565**	**0.554**	**0.525**
4. LSQ8: In terms of physical health, I am almost on a par with people my age.	0.243	**0.463**	0.316	−0.395	0.359	0.019	0.341	0.335	/
5. LSQ6: I sleep at least 7 to 8 h every night and wake up relaxed.	0.271	−0.015	0.267	0.123	0.158	0.230	0.237	/	/
6. LSQ3: I see a doctor regularly for medical examinations.	**0.418**	−0.138	**0.400**	0.280	0.060	**0.736**	/	/	/
7. LSQ7: Most of the time, I miss work due to illness.	0.141	−0.394	0.071	0.357	/	/	/	/	/
8. LSQ5: I have no chronic illnesses or physical disabilities.	0.084	**0.546**	/	/	/	/	/	/	/

Note. *N* = 200. The extraction method was principal axis factoring with an orthogonal (varimax) rotation. Factor loadings above 0.40 are in bold.

### Appendix A.3. Avoiding Drugs/Narcotics (PWB Indicator)

Exploratory factor analyses indicated good factorability (KMO = 0.74) with a one-factorial structure, accounting for 57% of the variance and factor loadings above the 0.400 threshold. The reliability was satisfactory (α = 0.88).

**Table A4:** Results from the factor analysis of the LSQ subscale avoiding drugs/narcotics.

Item	Factor Loading
1. LSQ50: I avoid taking drugs arbitrarily and indiscriminately.	**0.473**
2. LSQ51: I do not smoke.	**0.861**
3. LSQ52: I avoid drugs.	**0.849**
4. LSQ53: I avoid taking dangerous drugs and narcotics.	**0.846**
5. LSQ54: I avoid associating with addicts and alcoholics.	**0.676**
6. LSQ55: I avoid drinking alcohol.	**0.740**

Note. *N* = 200. The extraction method was principal axis factoring with an orthogonal (varimax) rotation. Factor loadings above 0.40 are in bold.

### Appendix A.4. Somatization (PD Indicator)

For the somatization subscale, exploratory factor analyses indicated good factorability (KMO = 0.74). According to the Eigenvalue criterion and parallel analysis, two factors could be extracted. When the items GHQ5 and GHQ7 were iteratively excluded, a one-factor solution emerged, accounting for 43% of the variance (α = 0.78).

**Table A5:** Results from the factor analysis of the GHQ-28 subscale somatization.

Item	Factor Loadings Based on Eigenvalue Criterion		Factor Loadings Based on Eigenvalue Criterion (EFA2)		Factor Loadings (EFA 3)
	Factor 1	Factor 2	Factor 1	Factor 2	Factor 1
1. Been feeling perfectly well and in good health?	**0.686**	0.058	**0.694**	**0.095**	**0.668**
2. Been feeling in need of a good tonic?	**0.645**	0.172	**0.647**	**0.192**	**0.676**
3. Been feeling run down and out of sorts?	**0.838**	0.184	**0.816**	**0.249**	**0.857**
4. Been feeling that you are ill?	**0.530**	0.303	**0.459**	**0.457**	**0.580**
5. Been getting a feeling of tightness or pressure in your head?	0.284	**0.663**	**0.335**	**0.373**	**0.442**
6. Been having hot or cold spells?	0.226	**0.448**	**0.069**	**0.805**	/
7. Been getting any pains in your head?	−0.022	**0.990**	/	/	/

Note. *N* = 200. The extraction method was principal axis factoring with an orthogonal (varimax) rotation. Factor loadings above 0.40 are in bold.

### Appendix A.5. Anxiety (PD Indicator)

Regarding the anxiety subscale, exploratory factor analyses indicated good factorability (KMO = 0.79), with a one-factorial structure accounting for 44% of the variance and factor loadings above 0.400. The reliability was satisfactory (α = 0.84).

**Table A6:** Results from the factor analysis of the GHQ-28 subscale anxiety.

Item	Factor Loading
8. Been losing much sleep over worry?	**0.668**
9. Been having difficulty in staying asleep once you fall asleep?	**0.516**
10. Been feeling constantly under strain?	**0.669**
11. Been getting edgy or bad tempered?	**0.755**
12. Been getting scared or panicky for no reason?	**0.601**
13. Been feeling everything is getting on top of you?	**0.708**
14. Been feeling nervous and strung-out all the time?	**0.673**

Note. *N* = 200. The extraction method was principal axis factoring with an orthogonal (varimax) rotation. Factor loadings above 0.40 are in bold.

### Appendix A.6. Social Dysfunction (PD Indicator)

For the social dysfunction subscale, exploratory factor analyses indicated good factorability (KMO = 0.81) with a one-factorial structure accounting for 43% of the variance. Factor loadings were sufficiently high, as was the scale’s reliability (α = 0.83).

**Table A7:** Results from the factor analysis of the GHQ-28 subscale social dysfunction.

Item	Factor Loading
15. Been managing to keep yourself busy and occupied?	**0.558**
16. Been taking longer over things you do?	**0.422**
17. Been feeling on the whole that you were doing things well?	**0.722**
18. Been satisfied with the way you have carried out your tasks?	**0.769**
19. Been feeling that you are playing a useful part in things?	**0.836**
20. Been feeling capable of making decisions about things?	**0.648**
21. Been able to enjoy your normal day-to-day activities?	**0.564**

Note. *N* = 200. The extraction method was principal axis factoring with an orthogonal (varimax) rotation. Factor loadings above 0.40 are in bold.

### Appendix A.7. Depression (PD Indicator)

Regarding the depression subscale, exploratory factor analyses indicated good factorability (KMO = 0.88), with a one-factorial structure accounting for 62% of the variance. Factor loadings were sufficiently high, as was the scale’s reliability (α = 0.92).

**Table A8:** Results from the factor analysis of the GHQ-28 subscale depression.

Item	Factor Loading
22. Been thinking of yourself as a worthless person?	**0.743**
23. Been feeling that life is entirely hopeless?	**0.792**
24. Been feeling that life is not worth living?	**0.869**
25. Been thinking of the possibility that you may do away with yourself?	**0.707**
26. Been feeling at times that you could not do anything because your nerves were too bad?	**0.731**
27. Been finding yourself wishing you were dead and away from it all?	**0.877**
28. Been finding that the idea of taking your own life keeps coming into mind?	**0.788**

Note. *N* = 200. The extraction method was principal axis factoring with an orthogonal (varimax) rotation. Factor loadings above 0.40 are in bold.

### Appendix A.8. Physical Activity

Exploratory factor analyses indicated good factorability (KMO = 0.84) with a one-factorial structure. However, reliability improved considerably when excluding the comparatively low-loading items *LSQ9*, *LSQ12*, and *LSQ15* (successively: α = 0.89; 0.90; 0.90; 0.92), as did the explained variance (from 55% to 74%). Looking at item wording, it became clear that those items describe affective/symptomatic states, while the other items refer to behavior. Thus, the shorter four-item scale was preferred.

**Table A9:** Results from the factor analysis of the LSQ subscale physical activity.

Item	Factor Loading	Factor Loading (Excl. LSQ9)	Factor Loading (Excl. LSQ12)	Factor Loading (Excl. LSQ15)
LSQ10: I do muscle exercises and strengthening at least several times per week.	**0.784**	**0.775**	**0.774**	**0.798**
LSQ11: I do vigorous exercise for at least 30 min a day, three times a week, such as walking, bodybuilding, or aerobics.	**0.876**	**0.887**	**0.913**	**0.938**
LSQ13: I do vigorous physical activity for at least 30 min a day, such as brisk walking.	**0.853**	**0.881**	**0.897**	**0.875**
LSQ14: I spend most of my free time exercising or doing physical activities such as cycling, walking, swimming, and other sports.	**0.856**	**0.854**	**0.859**	**0.832**
LSQ15: I feel healthy.	**0.652**	**0.633**	**0.578**	/
LSQ12: I have the energy to spend a day without feeling tired.	**0.643**	**0.628**	/	/
LSQ9: I have good physical resistance.	**0.437**	/	/	/

Note. *N* = 200. The extraction method was principal axis factoring with an orthogonal (varimax) rotation. Factor loadings above 0.40 are in bold.

### Appendix A.9. Fear (Negative Affect Indicator)

Regarding the subscale *fear*, exploratory factor analyses indicated good factorability (KMO = 0.79) but low reliability (α = 0.65). Based on the Eigenvalue criterion and parallel analysis, two factors could be extracted. The item jittery had a negative correlation (r = −0.431 ***) with the semantically similar item nervous (and all other items). We suspected that respondents did not understand it semantically, as was intended. Being medical students, they could have thought about jittering as a physical symptom, rather than as a synonym for nervousness. Upon excluding the item, the remaining five items (KMO = 0.79) were loaded on one factor (loadings > 0.400), accounting for 49% of variance and forming a reliable scale (α = 0.81).

**Table A10:** Results from the factor analysis of the PANAS-X subscale fear.

Item	Factor Loading
1. Afraid	**0.837**
2. Scared	**0.823**
3. Frightful	**0.706**
4. Nervous	**0.528**
5. Shaky	**0.543**

Note. *N* = 200. The extraction method was principal axis factoring with an orthogonal (varimax) rotation. Factor loadings above 0.40 are in bold.

### Appendix A.10. Guilt (Negative Affect Indicator)

Regarding the subscale *guilt*, exploratory factor analyses indicated good factorability (KMO = 0.82), with a one-factorial structure accounting for 51% of the variance. As per a loading below the 0.400 threshold (*ashamed*: 0.339), one item was excluded, whereby good factorability remained (KMO = 0.81), and the reliability improved (from 0.84 to 0.88). The remaining items exhibited adequately sized factor loadings. The extracted factor accounted for 59% of variance.

**Table A11:** Results from the factor analysis of the PANAS-X subscale guilt.

Item	Factor Loading
1. Guilty	**0.719**
2. Blameworthy	**0.855**
3. Angry at self	**0.690**
4. Disgusted with self	**0.775**
5. Dissatisfied with self	**0.788**

Note. *N* = 200. The extraction method was principal axis factoring with an orthogonal (varimax) rotation. Factor loadings above 0.40 are in bold.

### Appendix A.11. Hostility (Negative Affect Indicator)

Regarding the subscale *hostility*, exploratory factor analyses indicated good factorability (KMO = 0.82), with a one-factorial structure accounting for 51% of the variance. Reliability was adequate (α = 0.85), and factor loadings were above the 0.400 threshold.

**Table A12:** Results from the factor analysis of the PANAS-X subscale hostility.

Item	Factor Loading
1. Angry	**0.792**
2. Hostile	**0.687**
3. Irritable	**0.775**
4. Scornful	**0.433**
5. Disgusted	**0.719**
6. Loathing	**0.799**

Note. *N* = 200. The extraction method was principal axis factoring with an orthogonal (varimax) rotation. Factor loadings above 0.40 are in bold.

### Appendix A.12. Sadness (Negative Affect Indicator)

Regarding the subscale *sadness*, exploratory factor analyses indicated good factorability (KMO = 0.83), with a one-factorial structure accounting for 58% of the variance. Reliability was adequate (α = 0.87), and factor loadings exceeded the 0.400 threshold.

**Table A13:** Results from the factor analysis of the PANAS-X subscale sadness.

Item	Factor Loading
1. Sad	**0.786**
2. Blue	**0.798**
3. Lonely	**0.723**
4. Alone	**0.756**
5. Downhearted	**0.733**

Note. *N* = 200. The extraction method was principal axis factoring with an orthogonal (varimax) rotation. Factor loadings above 0.40 are in bold.

### Appendix A.13. Attentiveness (Positive Affect Indicator)

Regarding the subscale *attentiveness*, exploratory factor analyses indicated good factorability (KMO = 0.76), with a one-factorial structure accounting for 56% of the variance. The reliability was sufficiently high (α = 0.83), as were the factor loadings.

**Table A14:** Results from the factor analysis of the PANAS-X subscale attentiveness.

Item	Factor Loading
1. Concentrating	**0.876**
2. Attentive	**0.781**
3. Determined	**0.673**
4. Alert	**0.633**

*Note. N* = 200. The extraction method was principal axis factoring with an orthogonal (varimax) rotation. Factor loadings above 0.40 are in bold.

### Appendix A.14. Joviality (Positive Affect Indicator)

Regarding the subscale *joviality*, exploratory factor analyses indicated good factorability (KMO = 0.91). One factor emerged that accounted for 56% of the variance. The reliability was sufficiently high (α = 0.80), as were the factor loadings. However, the item lively exhibited a negative loading and correlated negatively with the remaining semantically similar items. When excluding it from the item pool (KMO = 0.92), one factor emerged, accounting for 61% of the variance. The scale reliability was high (α = 0.91).

**Table A15:** Results from the factor analysis of the PANAS-X subscale joviality.

Item	Factor Loading
1. Happy	**0.885**
2. Joyful	**0.845**
3. Energetic	**0.823**
4. Cheerful	**0.814**
5. Enthusiastic	**0.772**
6. Delighted	**0.754**
7. Excited	**0.540**

Note. *N* = 200. The extraction method was principal axis factoring with an orthogonal (varimax) rotation. Factor loadings above 0.40 are in bold.

### Appendix A.15. Self-Assurance (Positive Affect Indicator)

Regarding the subscale *self-assurance*, exploratory factor analyses indicated good factorability (KMO = 0.89), with a one-factorial structure accounting for 57% of the variance. The reliability was sufficiently high (α = 0.89), as were the factor loadings.

**Table A16:** Results from the factor analysis of the PANAS-X subscale self-assurance.

Item	Factor Loading
1. Proud	**0.692**
2. Strong	**0.751**
3. Confident	**0.669**
4. Bold	**0.861**
5. Daring	**0.763**
6. Fearless	**0.801**

Note. *N* = 200. The extraction method was principal axis factoring with an orthogonal (varimax) rotation. Factor loadings above 0.40 are in bold.

## Appendix B. Post-Hoc Power Analyses

This appendix contains additional information on post hoc conducted power analyses. Table A17 and Table A18 below depict results of post hoc power analyses emergent from the estimated SEM models depicted in Figure A1. Figure A16 and Figure A17 further show the respective emergent power curves. As it became clear, sufficient statistical power (around 0.80) can be assumed for some of the found effects but not for others. Thus, sample size-related limitations are present. For further information, the detailed step-by-step description provided within Wang and Rhemtulla [50] is recommended.

**Table A17:** Results from the post hoc power analysis of the SEM model shown in panel A) of Figure 1 by investigated effect.

Investigated (In)direct Effect	Statistical Power (*N* = 1000 Bootstrap Repetitions)
**Physical activity –> Positive Affect (b1)**	**0.796**
**Physical activity –> Negative Affect (b2)**	**0.761**
**Physical activity –> PWB (b3)**	**0.433**
**Positive Affect –> PWB (b4)**	**0.433**
**Negative Affect –> PWB (b5)**	**0.433**
**Physical activity –> Positive Affect –> PWB (ind1)**	**0.226**
**Physical activity –> Negative Affect –> PWB (ind2)**	**0.292**

Note. *N* = 200. Statistical power was calculated via the application supplied by Wang and Rhemtulla [50]. Limitations apply as mentioned by the accompanying research paper.

**Table A18:** Results from the post hoc power analysis of the SEM model shown in panel B of Figure 1 by investigated effect.

Investigated (In)direct Effect	Statistical Power (*N* = 1000 Bootstrap Repetitions)
**Physical activity –> Positive Affect (b1)**	**0.793**
**Physical activity –> Negative Affect (b2)**	**0.770**
**Physical activity –> PD (b3)**	**0.579**
**Positive Affect –> PD (b4)**	**0.579**
**Negative Affect –> PD (b5)**	**0.579**
**Physical activity –> Positive Affect –> PD (ind1)**	**0.336**
**Physical activity –> Negative Affect –> PD (ind2)**	**0.457**

Note. *N* = 200. Statistical power was calculated via the application supplied by Wang and Rhemtulla [50]. Limitations apply as mentioned by the accompanying research paper.
